# Evaluating Alternate Methods of Determining the Antimicrobial Efficacy of Contact Lens Care Products against Acanthamoeba Trophozoites

**DOI:** 10.3390/pathogens10020126

**Published:** 2021-01-27

**Authors:** Allison Campolo, Paul Shannon, Monica Crary

**Affiliations:** Alcon Research, LLC, Fort Worth, TX 76134, USA; allison.campolo@alcon.com (A.C.); stephen.shannon@alcon.com (P.S.)

**Keywords:** *Acanthamoeba*, contact lens care, propidium iodide, antimicrobial efficacy

## Abstract

*Acanthamoeba* keratitis (AK) is a serious ocular infection caused by a ubiquitous free-living amoeba, *Acanthamoeba*. This infection often results in extensive corneal damage and blindness, and is notoriously difficult to cure. While *Acanthamoeba* is an abundant organism, AK is most associated with contact lens hygiene noncompliance and inadequate contact lens care (CLC) disinfection regimens. Thus, accurate and timely antimicrobial efficacy testing of CLC solutions is paramount. Published methods for antimicrobial efficacy testing of *Acanthamoeba* trophozoites requires 14 days for results. Presently, alternate and/or rapid methods for evaluating CLC products rarely demonstrate equivalent results compared to commonly-reported methods. Propidium iodide is a cellular stain that can only bind to cells with damaged outer membranes. We evaluated propidium iodide staining as an alternative method for determining the relative antimicrobial efficacy of 11 different CLC products against *Acanthamoeba* trophozoites. Following exposure to a CLC product, the fluorescence intensity of propidium iodide in an *Acanthamoeba* population demonstrated a strong correlation to the log reduction determined by established, growth-based *Acanthamoeba* testing used to evaluate the antimicrobial efficacy of CLC products. Thus, propidium iodide was found to be an effective rapid tool for determining cell death in *Acanthamoeba* trophozoites following exposure to CLC solutions.

## 1. Introduction

As antimicrobial disinfection efficacy of contact lens care (CLC) solutions is critical, various disinfecting solutions have been developed over the years to ensure contact lenses can be worn safely and comfortably following cleaning and disinfection. These contact lens disinfecting solutions typically include antimicrobial biocides as well as cleaning, wetting, and disinfecting agents. For all of these solutions, the goal is to maintain sufficient antimicrobial activity so that the numbers of potentially pathogenic microorganisms are reduced substantially during the disinfectant exposure period.

*Acanthamoeba* keratitis (AK) is an eye infection caused by a free-living amoeba, *Acanthamoeba*, which can cause extensive corneal damage and often blindness [1]. Outbreaks of *Acanthamoeba* keratitis in the United States in 2007 [2] and the United Kingdom beginning in 2010 [3] have led to hundreds of infected patients, as well as the products or disinfectants associated with those outbreaks being recalled or phased out [2,3,4,5]. Critically, within populations of infected patients, as many as 41% of AK sufferers have become blind due to corneal damage [6]. Following this, the differences in *Acanthamoeba* disinfection between CLC products available to consumers, largely due to the biocides used in each product, continues to be a risk factor for the development of AK. Despite the specific quantity or concentrations of biocides in any particular CLC product, *Acanthamoeba* trophozoites and cysts are unique among pathogenic microorganisms and may be highly resistant to biocides which are effective against other pathogens.

Currently, the United States Food and Drug Administration and other international regulatory bodies require contact lens care solution manufacturers to conduct microbiological testing of CLC solution disinfection against fungi (*Fusarium keratoplasticum*), yeast (*Candida albicans*), and bacteria (*Pseudomonas aeruginosa*, *Serratia marcescens*, and *Staphylococcus aureus*) during product registration due to these organisms often being associated with potential ocular infections [7]. Unfortunately, these requirements still do not include *Acanthamoeba*; however, efforts are underway to make this critical microorganism part of the product registration requirements [8]. Thus, testing for *Acanthamoeba* is not uniformly addressed among CLC solution manufacturers, and current methods can be considered inefficient and time-consuming. Widely reported, growth-based testing methods, as well as recently-described novel methods that deviate from traditional testing, often require a 14-day incubation of *Acanthamoeba* following exposure of trophozoites to CLC products [9,10,11,12,13,14]. In general, these methods rely on the 14-day growth period to allow surviving *Acanthamoeba* to proliferate following exposure of a population to CLCs. From this, the 50% endpoint can be calculated. However, these methods can be inherently lengthy and are often described as percent viability of the number of trophozoites present after CLC exposure, which cannot be correlated to the published growth-based log reduction results. Presently, alternate and/or rapid methods for evaluating CLC products rarely demonstrate equivalent results compared to commonly-reported methods [15]. Thus, we here describe a new method for *Acanthamoeba* testing of CLC solutions, which is both expedient and accurate, in direct comparison to the more commonly used methods, and which may serve to improve *Acanthamoeba* testing industry-wide for CLC solution manufacturers.

## 2. Results

### 2.1. Acanthamoeba Quantification Using Published Growth-Based Methods

**Figure 1:**
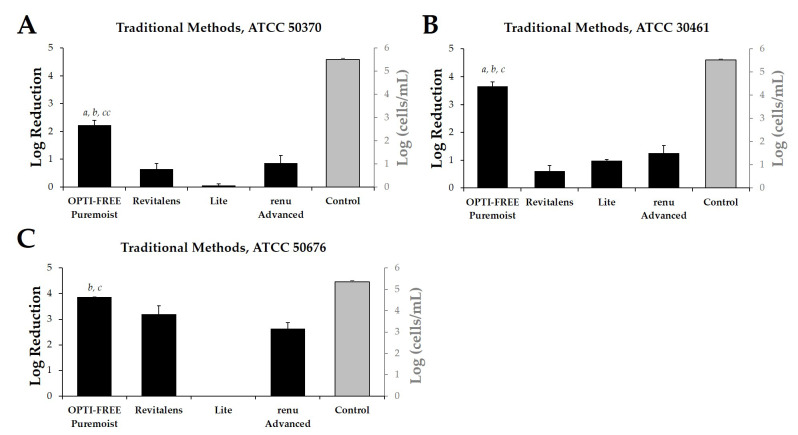
Log reduction from growth-based method testing with *Acanthamoeba* trophozoites following exposure to four contact lens care (CLC) products. Left y-axis, in black: mean ± SE quantification of log reduction (difference of control cells/mL and CLC-treated cells/mL) of *Acanthamoeba* strain (**A**) ATCC 50370, (**B**) ATCC 30461, and (**C**) ATCC 50676 following CLC exposure. Right y-axis, in grey: control cells/mL (untreated *Acanthamoeba* culture grown in tandem with treated cultures). N = 6/group; *a p* < 0.001 vs. Revitalens, *b p* < 0.001 vs. Lite, *cc p* < 0.01 vs. renu Advanced, *c p* < 0.001 vs. renu Advanced.

Following the widely reported, growth-based method requiring 14-day incubation of three separate *Acanthamoeba* strains (ATCC 50370 (*Acanthamoeba castellani*), 30461 (*Acanthamoeba polyphaga*), and 50676 (*Acanthamoeba mauriteniensis*); Table 1), we determined the log reduction of *Acanthamoeba* after exposure to CLC solutions using the manufacturer-recommended disinfection time of each product (Figure 1A–C). OPTI-FREE Puremoist Multi-Purpose Disinfecting Solution (MPDS) demonstrated greater than 2 log reduction against each of the tested *Acanthamoeba* strains examined. OPTI-FREE Puremoist MPDS also demonstrated a significantly higher (*p* < 0.01) log reduction of all three *Acanthamoeba* spp. compared to other CLC products tested, using growth-based methods (Figure 1A–C).

### 2.2. Experimental Method Development

**Figure 2:**
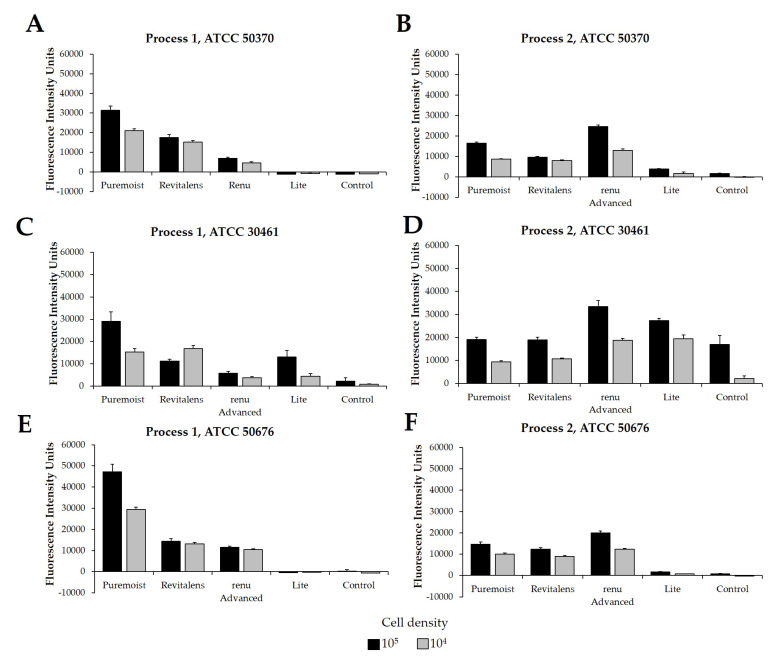
Fluorescence intensity of propidium iodide staining with Process 1 and Process 2. Mean ± SE of fluorescence intensity units. Process 1 results using *Acanthamoeba* strain (**A**) ATCC 50370, (**C**) ATCC 30461, and (**E**) ATCC 50676. Process 2 results using *Acanthamoeba* strain (**B**) ATCC 50370, (**D**) ATCC 30461, and (**F**) ATCC 50676. N = 9/group. Grey bars indicate a cell density of 1 × 10^4^ per well, and black bars indicate a cell density of 1 × 10^5^ per well.

**Figure 3:**
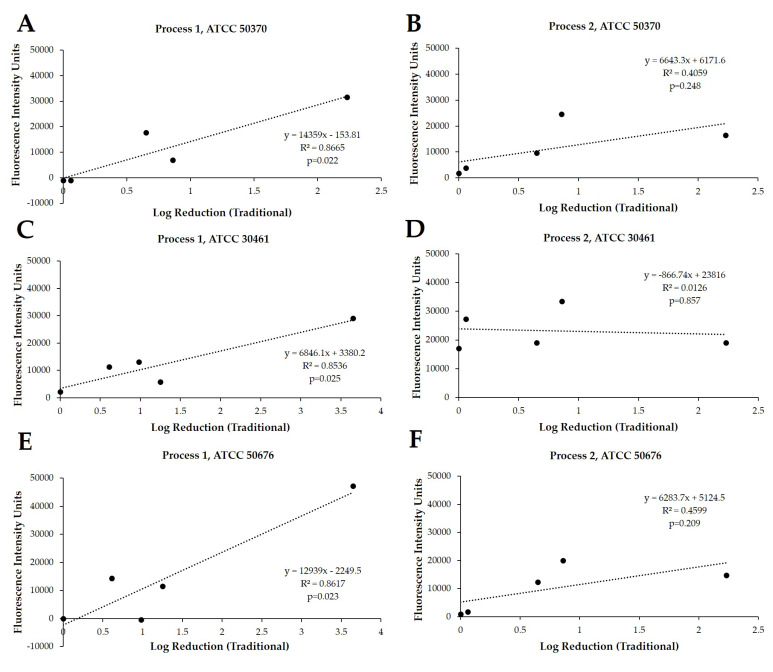
Significant linear correlation between growth-based recovery and Process 1, but not with Process 2. Traditional method results (log reduction) and Process 1 (fluorescence intensity units) correlation using *Acanthamoeba* strain (**A**) ATCC 50370, (**C**) ATCC 30461, and (**E**) ATCC 50676, and Process 2 correlation using *Acanthamoeba* strain (**B**) ATCC 50370, (**D**) ATCC 30461, and (**F**) ATCC 50676. From each Process, the cell density of 1 × 10^5^ was used to calculate correlation. N = 6–9 replicates/point.

**Figure 4:**
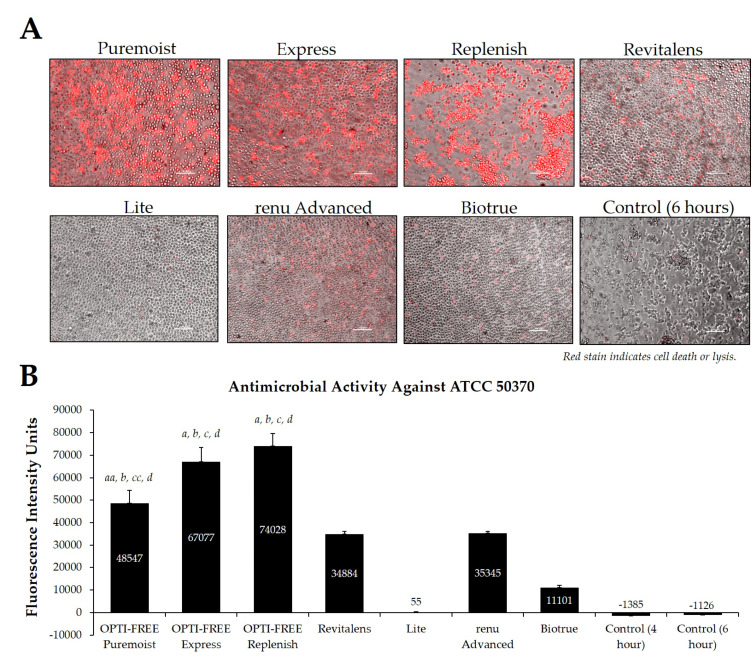
Fluorescence intensity of propidium iodide staining with *Acanthamoeba* strain ATCC 50370 following exposure to seven CLC products. (**A**) Representative images of fluorescent propidium iodide staining using 10× magnification, followed by (**B**) associated mean ± SE quantification of fluorescence intensity units. N = 15/group; *aa p* < 0.01 vs. Revitalens, *a p* < 0.001 vs. Revitalens, *b p* < 0.001 vs. Lite, *cc p* < 0.01 vs. renu Advanced, *c p* < 0.001 vs. renu Advanced, and *d p* < 0.001 vs. Biotrue. Red stain indicates dead or lysed cells, and grey cells are living at the time of analysis. Cell density = 1 × 10^5^, scale bar = 100 µm. Control samples indicate untreated *Acanthamoeba* wells.

**Figure 5:**
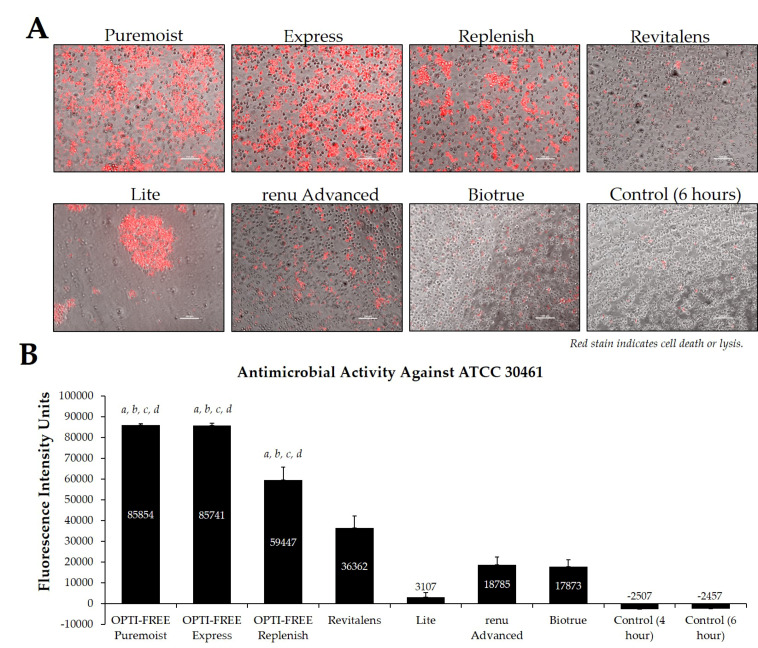
Fluorescence intensity of propidium iodide staining with *Acanthamoeba* strain ATCC 30461 following exposure to seven CLC products. (**A**) Representative images of fluorescent propidium iodide staining using 10× magnification, followed by (**B**) associated mean ± SE quantification of fluorescence intensity units. N = 15/group; *a p* < 0.001 vs. Revitalens, *b p* < 0.001 vs. Lite, *c p* < 0.001 vs. renu Advanced, and *d p* < 0.001 vs. Biotrue. Red stain indicates dead or lysed cells, and grey cells are living at the time of analysis. Cell density = 1 × 10^5^, scale bar = 100 µm. Control samples indicate untreated *Acanthamoeba* wells.

**Figure 6:**
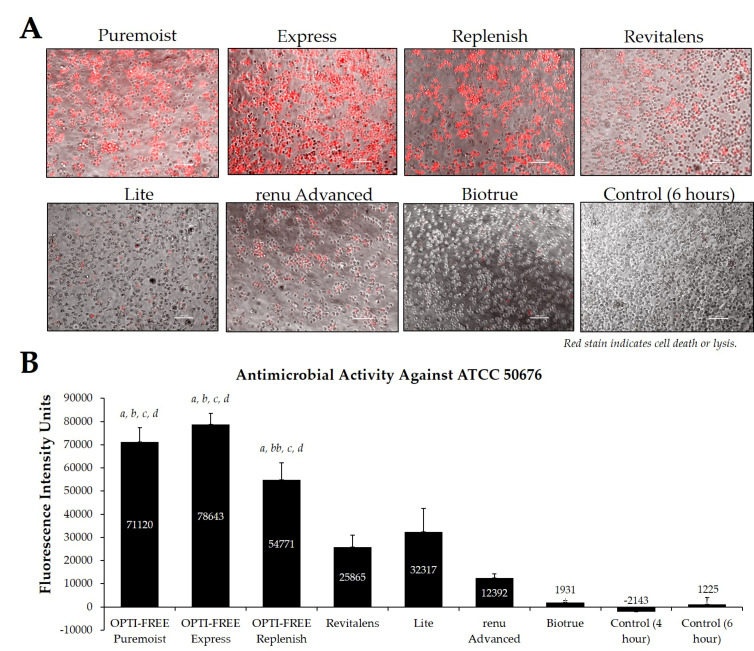
Fluorescence intensity of propidium iodide staining with *Acanthamoeba* strain ATCC 50676 following exposure to seven CLC products. (**A**) Representative images of fluorescent propidium iodide staining using 10× magnification, followed by (**B**) associated mean ± SE quantification of fluorescence intensity units. N = 15/group; *a p* < 0.001 vs. Revitalens, *bb p* < 0.01 vs. Lite, *b p* < 0.001 vs. Lite, *c p* < 0.001 vs. renu Advanced, and *d p* < 0.001 vs. Biotrue. Red stain indicates dead or lysed cells, and grey cells are living at the time of analysis. Cell density = 1 × 10^5^, scale bar = 100 µm. Control samples indicate untreated *Acanthamoeba* wells.

**Figure 7:**
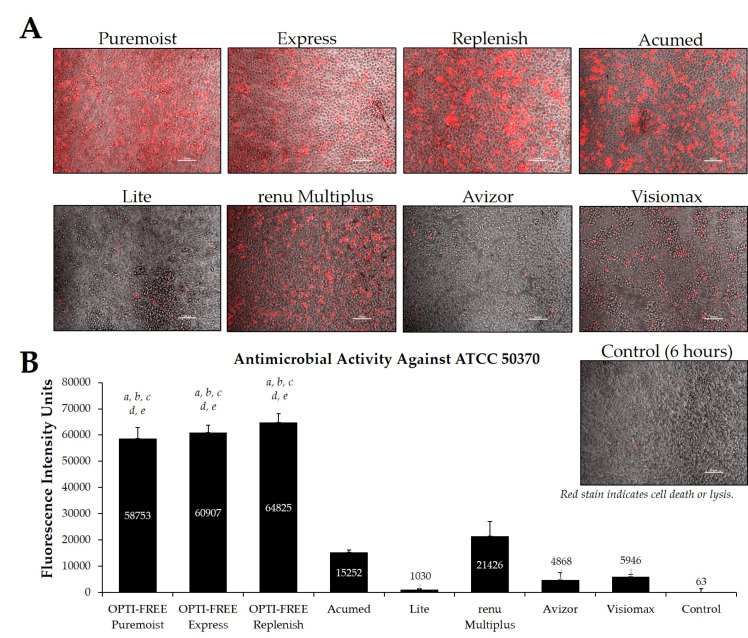
Fluorescence intensity of propidium iodide staining with *Acanthamoeba* strain ATCC 50370 following exposure to eight CLC products in the European market. (**A**) Representative images of fluorescent propidium iodide staining using 10× magnification, followed by (**B**) associated mean ± SE quantification of fluorescence intensity units. N = 15/group; *a p* < 0.001 vs. Acumed, *b p* < 0.001 vs. Lite, *c p* < 0.001 vs. renu Multiplus, *d p* < 0.001 vs. Avizor, and *e p* < 0.01 vs. Visiomax. Red stain indicates dead or lysed cells, and grey cells are living at the time of analysis. Cell density = 1 × 10^4^, scale bar = 100 µm. Control samples indicate untreated *Acanthamoeba* wells at 6 h.

**Figure 8:**
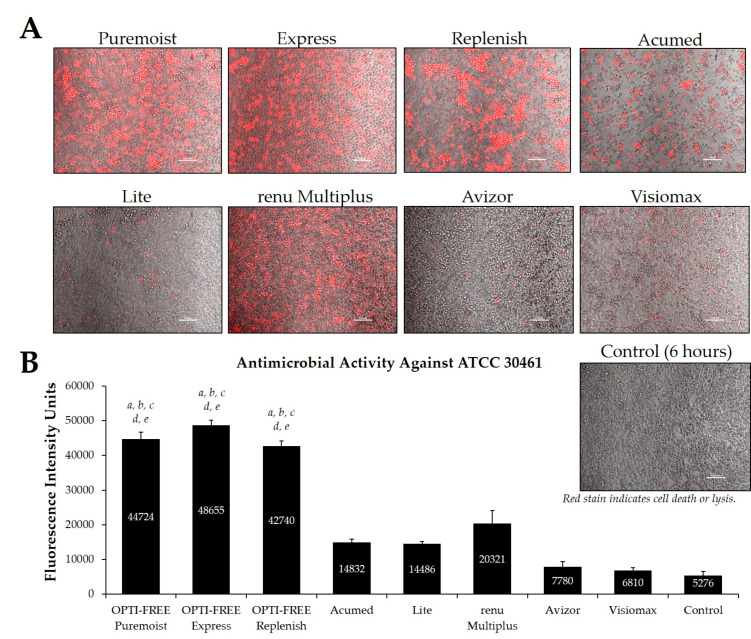
Fluorescence intensity of propidium iodide staining with *Acanthamoeba* strain ATCC 30461 following exposure to eight CLC products in the European market. (**A**) Representative images of fluorescent propidium iodide staining using 10× magnification, followed by (**B**) associated mean ± SE quantification of fluorescence intensity units. N = 15/group; *a p* < 0.001 vs. Acumed, *b p* < 0.001 vs. Lite, *c p* < 0.001 vs. renu Multiplus, *d p* < 0.001 vs. Avizor, and *e p* < 0.01 vs. Visiomax. Red stain indicates dead or lysed cells, and grey cells are living at the time of analysis. Cell density = 1 × 10^4^, scale bar = 100 µm. Control samples indicate untreated *Acanthamoeba* wells at 6 h.

Following this study, the same *Acanthamoeba* strain and CLC products were challenged using two different experimental protocols, Process 1 (Figure 2A,C,E) and Process 2 (Figure 2B,D,F), using cellular densities of 1 × 10^4^ to 5 × 10^5^ *Acanthamoeba* trophozoites. These processes were quantified in fluorescence intensity units (FIU) by comparing CLC solutions to blanks and internal controls, as well as to each other, when run on the same plate and read at the same time. These FIU quantifications were then compared to the log reduction quantification of widely reported testing methods via linear correlation (Figure 3). For correlation and for the following figures (Figure 3, Figure 4, Figure 5, Figure 6, Figure 7 and Figure 8), the lowest cell density that demonstrated a differentiation between products was used. Process 1 FIU results were found to have a significant correlation (*p* < 0.05) with the traditional log reduction results (Figure 3A,C,E) while Process 2 FIU results were found to have no correlation with traditional log reduction outcomes (Figure 3B,D,F). Together, these results indicated that the novel Process 1 is highly indicative of, and representative of, growth-based *Acanthamoeba* quantification methods. Therefore, the Process 1 method was chosen for the broader testing of a wide-range of CLC solutions.

### 2.3. Testing Representative Solutions Available from United States Manufacturers, Using Process 1

Using CLC products from representative United States manufacturers, we then examined the *Acanthamoeba* strain ATCC 50370 (Figure 4), ATCC 30461 (Figure 5) (as the two most commonly used strains in CLC product testing), and ATCC 50676 (Figure 6) (a more novel strain) using the Process 1 method [8,16]. Images were taken of the fluorescence of each well (subpanel A), and quantifications of each 96-well plate were calculated using a BioTek plate reader (subpanel B). In all three strains, OPTI-FREE Puremoist MPDS, OPTI-FREE Express MPDS, and OPTI-FREE Replenish MPDS, demonstrated significantly higher FIU (*p* < 0.01, indicating greater amoebic death based on greater amount of red fluorescent stain) than Revitalens, Lite, renu Advanced, or Biotrue. In some CLC solutions tested, cell lysis occurred and propidium iodide was still able to bind to exposed cellular contents. The staining of these cellular components can make the media appear to possess a red tinge or over-fluorescence, despite the dye still binding to exposed *Acanthamoeba* cellular DNA.

### 2.4. Testing Solutions in the European Market, Using Process 1

Additionally, CLC solutions sold in the European market were investigated against the *Acanthamoeba* strains ATCC 50370 (Figure 7) and ATCC 30461 (Figure 8) using the Process 1 method, as they are the two most widely tested strains in CLC product research [8,16]. In both strains, OPTI-FREE Puremoist MPDS, OPTI-FREE Express MPDS, and OPTI-FREE Replenish MPDS demonstrated significantly higher FIU (*p* < 0.001) than Acumed, Lite, renu Multiplus, Avizor, or Visiomax. Similar to the CLC solutions from representative U.S. manufacturers, cell lysis occurred in some solutions, and propidium iodide is still able to bind to exposed cellular contents. The staining of these cellular components can make the media appear to possess a red tinge or over-fluorescence, despite the dye still binding to exposed *Acanthamoeba* cellular DNA.

## 3. Discussion

Contact lens care cases, even those used by asymptomatic lens wearers, have been found to have contamination by viable bacteria colonies and *Acanthamoeba* trophozoites or cysts [17]. Importantly, *Acanthamoeba* keratitis (AK) in particular is difficult to diagnose and can be extremely challenging to treat or manage, with potentially devastating effects to eye function [1,6]. The most direct risk factor for developing an AK infection continues to be improper use of CLC products or ineffective CLC products themselves [18]. Outbreaks of *Acanthamoeba* in the United States and the United Kingdom [2,3,4,5] point to the challenge of maintaining disinfected reusable contact lenses even among products used from established CLC solution manufacturers and in developed countries. Critical next steps in the prevention of AK outbreaks include the development of standardized methods for the culture of *Acanthamoeba* and rigorous testing of contact lens care systems that kill or disinfect *Acanthamoeba* from contact lenses [19]. In this endeavor, the FDA 510[k] and International Standards Organization 14729 do not yet include *Acanthamoeba* disinfection testing for CLC products, but efforts are underway to standardize and include such testing in the near future as a new standard. We believe the method for *Acanthamoeba* testing of CLC products described here will improve efficiency, expediency, and accuracy of testing, thus immediately improving the ability of CLC product manufacturers to accurately assess their products for antimicrobial disinfection efficacy.

Widely reported, growth-based testing methods, as well as recently-described novel methods that deviate from traditional testing, often require a 14-day incubation of *Acanthamoeba* following exposure of trophozoites to CLC products [9,10,11,12,13,14]. However, these methods can be inherently lengthy and are often described as percent viability of the number of trophozoites present after CLC exposure, which cannot be correlated to the published growth-based log reduction results. The novel method described here, which is also a high-throughput assay, not only shortens the traditional testing time by two weeks, but is immediately and directly quantifiable via plate reading of fluorescence. Additionally, to our knowledge, this is the first attempt at an *Acanthamoeba* disinfection method development among CLC solutions that demonstrates a direct and significant correlation between growth-based methods and the novel method being described. In this endeavor, we have analyzed the antimicrobial activity of a wider range of marketed CLC products than other recent method advancement attempts [12,14]. Further, this novel method development examines more individual strains of *Acanthamoeba* within one body of work, which are the most common pathogens associated with AK and are strains that are well-established laboratory and clinical strains thoroughly described in the literature [20,21,22], which is more than other method development attempts [12,14,15,23,24].

In exploring these novel methods, cell densities near 1 × 10^4^ and 1 × 10^5^ were used to scrutinize Processes 1 and 2 for quantitative rigor, and Pearson correlations [25] with traditional log reduction results were calculated using the 1 × 10^5^ cell densities. Critically, these correlations indicate a significant relationship between the Process 1 novel method and the growth-based methods used until now, and indicate that Process 1 can be used in place of the more cumbersome commonly used methods. Process 2, and potentially other attempts at rapid method development, demonstrated a lack of correlation with published methods due to the alteration of cellular permeability by CLC solutions. Cell permeability being impacted by the presence of the biocides within CLC solutions is well-established for corneal cells and would have equal impact on *Acanthamoeba* trophozoites [26]. Thus, Process 1′s clear correlation succeeds in part due to the removal of the CLC products immediately prior to the addition of the propidium iodide. The removal of CLCs allows *Acanthamoeba* trophozoites to stabilize their cell permeability in the absence of biocides and facilitates an appropriate uptake of propidium iodide by compromised cells to occur, while also preventing nonselective uptake by viable trophozoites in the presence of biocides. It should additionally be noted that the novel processes examined here will not be applicable to hydrogen peroxide-based CLC solutions due to the required H_2_O_2_ neutralization process. This neutralization process requires specific enzyme kinetics over the 6-h disinfection period within the designated lens care cup, and transferring this process to a 96-well plate for staining introduces inappropriate alterations to the activity of the CLC solution and/or to the *Acanthamoeba* quantification. Thus, this process has not yet been examined to assess *Acanthamoeba* cyst viability, as cysts are inherently far more difficult to eradicate due to their difference in cell walls (and efficacy against cysts requires H_2_O_2_-based disinfection systems). Similarly, novel rapid-method tests rely on dyes (including propidium iodide) that depend on the cellular permeability to indicate cell viability, and cyst cell walls may not allow for the uptake of these dyes appropriately. Due to these difficulties in accurately testing for and eradicating the cystic form of *Acanthamoeba*, one of the goals of many CLC solutions is to prevent the formation of cysts so that *Acanthamoeba* may remain in the more vulnerable trophozoite form [27,28]—a form to which all of the presently-examined CLCs would be applicable.

A major component of this novel quantitative method development is the propidium iodide staining. Previous investigations attempting to introduce novel *Acanthamoeba* quantification methods have depended upon Alamar Blue [15,29], which relies on the dye to be taken up by living cells in order to be accurately quantified. The data for the success of using this dye in *Acanthamoeba* (or indeed, protozoa in general) are extremely limited, and the ability of living *Acanthamoeba* trophozoites to preferentially refuse a dye cannot be ignored. Further, Trypan Blue staining methods depend solely upon a hemocytometer cell count, and thus, are less accurately quantified [30]. Additionally, it must be stated that staining methods that involve flow cytometry depend on cell permeability to dyes to facilitate accurate counting, which are difficult to accurately align with the use of flow and have failed to appropriately address trophozoite permeability in the presence of biocides [23,24,31]. Most importantly, to our knowledge, none of these methods can be or were directly compared to widely-reported testing methods to ensure an equal comparison, indicating a major flaw of method development so far.

The propidium iodide staining used here has been described as a suitable stain for amoeboid protist quantification in other recent investigations [32,33]. Propidium iodide is a common and ideal choice for the quantification of dead cells due to its inability to permeate cell membranes and due to its 20- to 30-fold increased fluorescence once the dye binds to DNA [31]. One of the detractions cited for the use of propidium iodide is the need for washing after application of the dye in order to reduce fluorescent oversaturation, which could remove the amoeba from a sample prior to quantification and/or provide inconsistent results of the staining method [34]. Thus, by diluting the dye to a final concentration of 2 µg/mL in the well and adding it immediately before quantification, the need for washing is eliminated, thereby retaining all living and dead organisms within the well and substantially reducing any potential variability of the staining method. Finally, propidium iodide is superior to other commonly used dead-cell stains, such as ethidium bromide, due to propidium iodide’s specific wavelength emission [31]. It can also be considered superior to ethidium homodimer (EthD-1) as EthD-1 is a harmful, toxic, and expensive dye, and propidium iodide has demonstrated widely similar quantification results as EthD-1 [35].

Following the Process 1 method, it was determined that the OPTI-FREE MPDS products, namely Puremoist, Express, and Replenish, maintained a consistently higher average of fluorescence intensity units, indicating greater *Acanthamoeba* trophozoite death than the other CLC products tested. These results were consistent among all three *Acanthamoeba* strains examined (ATCC 50370, ATCC 30461, and ATCC 50676), and among the CLC solutions tested in both the United States and European market groups. This difference can be attributed to the biocide differences between these products, as the OPTI-FREE MPDS contain 10 parts per million (ppm) of polyquaternium-1, compared to the other products containing only 1 to 3 ppm of similar biocides.

In conclusion, we here describe a novel *Acanthamoeba* trophozoite quantification method, which both reduces variability in the interpretation of results as well as saves a substantial amount of time in the endeavor of testing the *Acanthamoeba*-disinfecting properties of CLC products. This method further provides a novel option for high-throughput screening of CLC solutions in development to promote faster turnaround time. Overall, this method is highly consistent with the currently-used growth-based methods, and both methods indicate a significantly higher *Acanthamoeba* disinfection efficacy among the OPTI-FREE MPDS products versus the other CLC solutions tested.

## 4. Materials and Methods

### 4.1. Acanthamoeba Trophozoite Culturing for All Methods

Axenic culture media (AC6; containing 20 g biosate peptone, 5 g glucose, 0.3 KH_2_PO_4_, 10 µg vitamin B12, and 15 mg L-methionine per liter of distilled deionized water) was used to axenically produce *Acanthamoeba* trophozoites. AC6 media was adjusted to a pH of 6.6–6.95 with 1 M NaOH and autoclaved at 121 °C for 20 min before storing at room temperature for use within 3 months. One quarter Ringer’s solution was used to harvest organisms and for seeding trophozoites into 96-well plates.

### 4.2. Growth-Based Method for Quantification of Acanthamoeba Following Incubation in CLC Solutions

Antimicrobial efficacy of contact lens disinfecting solutions against *Acanthamoeba* trophozoites was conducted in a traditional manner, per a modified version of ISO standard 14729, as an internal control for the experimental methods developed here. *Acanthamoeba* strains ATCC 50370, 30461 and 50676 (Table 1, American Type Culture Collection, Manassas, VA, USA) were utilized for testing. These strains belong to the T4 genotype, which, while there have been several genotypes isolated from *Acanthamoeba* keratitis patients, is the most commonly associated genotype with this infection, accounting for at least 90% of all *Acanthamoeba* keratitis cases [36,37,38]. *Acanthamoeba* trophozoites were subcultured in axenic media with the final 24-h of growth done in fresh media to promote uniform *Acanthamoeba* trophozoite proliferation prior to testing to ensure a homogenous population of trophozoites. Trophozoites were inoculated into each CLC solution for a final cell density between 1 × 10^4^ and 5 × 10^5^ cells per well. Each CLC solution was held at room temperature for disinfection time (Table 2). At disinfection time, 1 mL of CLC solution was placed in 9 mL of neutralizing broth (lecithin and polysorbate 80) and serially diluted. Each dilution was plated in quadruplicate on a 12-well plate containing 2 mL of non-nutrient agar with 100 µL of *Escherichia coli* (10^8^ CFU/mL; ATCC 8739). Plates were incubated for 14 days at 28 ± 2 °C. After incubation, positive wells were identified and cell densities were determined using the 50% endpoint following the Reed and Muench computation [39]. Antimicrobial efficacy was determined by calculating the log reduction between the initial inoculum controls and the cell densities recovered from the CLC solution at disinfection time. Each *Acanthamoeba* strain was tested in two independent trials on different days and the results were averaged. All contact lens disinfecting solutions were tested simultaneously using the same inoculum stock as a direct comparison.

### 4.3. Experimental Quantitative Method Development

For method development, the two most-commonly used strains of *Acanthamoeba* trophozoites in CLC product testing (ATCC 50370, ATCC 30461) [8,16] and one more novel strain (ATCC 50676) were seeded into a black clear-bottom 96-well plate at a density of 1 × 10^4^ to 5 × 10^5^ cells per well using ¼ Ringer’s solution. Cells were allowed to adhere for two hours. Ringer’s was removed and CLC solutions (Table 2) were added to appropriate wells (0.2 mL/well) in nine replicates per cell density. Blanks and untreated *Acanthamoeba* wells were included as controls. Untreated wells additionally allowed for the observance of natural cell death in the face of no disinfectant challenge, as is evident in representative images (Figure 4, Figure 5, Figure 6, Figure 7 and Figure 8). *Acanthamoeba* 96-well plates were incubated according to the listed CLC manufacturer disinfection time.

Following this, two separate methodological processes were tested. For Process 1, CLC solutions were removed and propidium iodide (Invitrogen, Carlsbad, CA, USA) diluted in ¼ Ringer’s solution was added to each well. For Process 2, propidium iodide was added directly to each well containing CLC solution. For both processes, propidium iodide was diluted to a final concentration of 2 µg/mL within each well, which alleviated the need for a washing step. The 96-well plates were read for fluorescence intensity (544 nm/620 nm) on a BioTek Synergy Microplate reader (BioTek Instruments, Inc., Winooski, VT, USA) immediately following the addition of propidium iodide. The FIU of blank wells were subtracted from the control and experimental wells, which allowed for the possibility of negative FIU values. Due to the normalization steps involved in the calculation of FIU, these are expressed in relative units [40]. This was followed by confocal imaging (Nikon Ti Eclipse Microscope, Nikon, Minato City, Tokyo, Japan) using the Nikon NIS-Elements platform.

To reduce variation, all compared test solutions, untreated wells, and blanks were run on the same plates at the same time, and the same lot of propidium iodide was used for all experiments.

### 4.4. Use of Process 1 Method for Broad Testing of CLC Solutions

Process 1 was followed for the final assay parameters. For this, two standard laboratory strains of *Acanthamoeba* trophozoites (ATCC 50370 and ATCC 30461) and one standard clinical strain (ATCC 50676) were seeded into a black clear-bottom 96-well plate at a density of 1 × 10^4^ and 1 × 10^5^ cells per well. Cells were allowed to adhere for 2 h. Media were removed and CLC solutions were added to appropriate wells (0.2 mL/well) in 15 replicates. Blanks and untreated *Acanthamoeba* wells were included as controls. Following the CLC manufacturer’s listed disinfection time, CLC solutions were removed and 0.2 mL of propidium iodide diluted in ¼ Ringer’s solution (for a final propidium iodide concentration of 2 µg/mL) was added to each well. The 96-well plates were read for fluorescence intensity (544 nm/620 nm) on a Biotek microplate reader immediately following the addition of the propidium iodide solution, followed by confocal imaging. For each *Acanthamoeba* strain, the lowest density necessary to identify a differentiation between CLC products was used (specific density for each experiment are listed within figure legends).

### 4.5. Statistical Analysis

A student’s two-tailed t-test was performed following verification of equal variance for each comparison. Quantifications are presented as mean ± standard error. Correlations were performed by calculating a Pearson’s correlation coefficient. Significance was determined as *p* < 0.05.

## 5. Patents

PAT058670-US-PSP, filed 23 July 2020, submitted by Monica Crary, Paul Shannon, and Alcon Research, LLC.

## Figures and Tables

**Table 1:** Test organisms used, and their strain, group, and isolation source.

Test Microorganism	Species	Group	Isolation Source
Acanthamoeba castellanii trophozoites	ATCC 50370	T4	Human eye infection, New York, NY 1978
Acanthamoeba polyphaga trophozoites	ATCC 30461	T4	Human corneal scrapings, Houston, TX, 1973
Acanthamoeba mauritaniensis trophozoites	ATCC 50676	T4	Human eye infection, Namibia or South Africa, 1990

**Table 2:** Contact lens care products used, and their manufacturers, biocides, and stated disinfection times.

Contact Lens Care Product	Manufacturer	Biocides	Disinfection Time
OPTI-FREE® Puremoist^®^	Alcon^®^	polyquaternium-1 (0.001%), myristamidopropyl dimethylamine (0.0006%)	6 h
OPTI-FREE® Express^®^	Alcon^®^	polyquaternium-1 (0.001%), myristamidopropyl dimethylamine (0.0005%)	6 h
OPTI-FREE® Replenish^®^	Alcon^®^	polyquaternium-1 (0.001%), myristamidopropyl dimethylamine (0.0005%)	6 h
Acuvue™ Revitalens^®^	Johnson & Johnson	polyquaternium-1 (0.0003%), alexidine dihydrochloride (0.00016%)	6 h
renu® Advanced Formula	Bausch + Lomb	polyquaternium (0.00015%), alexidine dihydrochloride (0.0002%), polyaminopropyl biguanide (0.00005%)	4 h
Biotrue^®^	Bausch + Lomb	polyaminopropyl biguanide (0.00013%), polyquaternium (0.0001%)	4 h
Lite™	CooperVision	polyhexanide (0.0001%)	6 h
Kombi-Clean & Moist	Acumed^®^	polyhexamethylene biguanide (0.0002%), polyquaternium (0.004%)	6 h
All Clean® Soft	Avizor	polyhexanide (0.0002%)	4 h
Kombilösung Super	Visiomax^®^	polyhexamethylene biguanide (0.0002%)	4 h
renu® Multiplus^®^	Bausch + Lomb	polyhexamethylene biguanide (0.0001%)	4 h

## Data Availability

The data presented in this study are available upon request from the corresponding author. The data are not publicly available due to commercial interests.

## References

[1] Szentmary N., Daas L., Shi L., Laurik K.L., Lepper S., Milioti G., Seitz B. (2019). Acanthamoeba keratitis‑clinical signs, differential diagnosis and treatment. J. Curr. Ophthalmol..

[2] Verani J.R., Lorick S.A., Yoder J.S., Beach M.J., Braden C.R., Roberts J.M., Conover C.S., Chen S., McConnell K.A., Chang D.C. (2009). National outbreak of acanthamoeba keratitis associated with use of a contact lens solution, united states. Emerg. Infect. Dis..

[3] Carnt N., Hoffman J.J., Verma S., Hau S., Radford C.F., Minassian D.C., Dart J.K.G. (2018). Acanthamoeba keratitis: Confirmation of the uk outbreak and a prospective case-control study identifying contributing risk factors. Br. J. Ophthalmol..

[4] Datta A., Willcox M.D.P., Stapleton F. (2020). In vivo efficacy of silver-impregnated barrel contact lens storage cases. Contact Lens Anterior Eye.

[5] Tu E.Y., Joslin C.E. (2010). Recent outbreaks of atypical contact lens-related keratitis: What have we learned?. Am. J. Ophthalmol..

[6] Scruggs B.A., Quist T.S., Salinas J.L., Greiner M.A. (2019). Notes from the field: Acanthamoeba keratitis cases-iowa, 2002–2017. MMWR Morb. Mortal. Wkly. Rep..

[7] International Standards Organization ISO 14729. https://www.iso.org/standard/25382.html.

[8] Brocious J., Tarver M.E., Hampton D., Eydelman M. (2018). Acanthamoeba: An overview of the challenges to the development of a consensus methodology of disinfection efficacy testing for contact lens care products. Eye Contact Lens.

[9] Niszl I.A., Markus M.B. (1998). Anti-acanthamoeba activity of contact lens solutions. Br. J. Ophthalmol..

[10] Hughes R., Kilvington S. (2001). Comparison of hydrogen peroxide contact lens disinfection systems and solutions against acanthamoeba polyphaga. Antimicrob. Agents Chemother..

[11] Hiti K., Walochnik J., Haller-Schober E.M., Faschinger C., Aspock H. (2002). Viability of acanthamoeba after exposure to a multipurpose disinfecting contact lens solution and two hydrogen peroxide systems. Br. J. Ophthalmol..

[12] Kilvington S., Lam A. (2013). Development of standardized methods for assessing biocidal efficacy of contact lens care solutions against acanthamoeba trophozoites and cysts. Investig. Ophthalmol. Vis. Sci..

[13] Johnston S.P., Sriram R., Qvarnstrom Y., Roy S., Verani J., Yoder J., Lorick S., Roberts J., Beach M.J., Visvesvara G. (2009). Resistance of acanthamoeba cysts to disinfection in multiple contact lens solutions. J. Clin. Microbiol..

[14] Kolar S.S., Manarang J.C., Burns A.R., Miller W.L., McDermott A.M., Bergmanson J.P. (2015). Contact lens care solution killing efficacy against acanthamoeba castellanii by in vitro testing and live-imaging. Contact Lens Anterior Eye.

[15] McBride J., Ingram P.R., Henriquez F.L., Roberts C.W. (2005). Development of colorimetric microtiter plate assay for assessment of antimicrobials against acanthamoeba. J. Clin. Microbiol..

[16] Marciano-Cabral F., Cabral G. (2003). Acanthamoeba spp. As agents of disease in humans. Clin. Microbiol. Rev..

[17] Larkin D.F., Kilvington S., Easty D.L. (1990). Contamination of contact lens storage cases by acanthamoeba and bacteria. Br. J. Ophthalmol..

[18] Radford C.F., Minassian D.C., Dart J.K. (2002). Acanthamoeba keratitis in england and wales: Incidence, outcome, and risk factors. Br. J. Ophthalmol..

[19] Carnt N., Stapleton F. (2016). Strategies for the prevention of contact lens-related acanthamoeba keratitis: A review. Ophthalmic Physiol. Opt..

[20] Thomson S., Rice C.A., Zhang T., Edrada-Ebel R., Henriquez F.L., Roberts C.W. (2017). Characterisation of sterol biosynthesis and validation of 14alpha-demethylase as a drug target in acanthamoeba. Sci. Rep..

[21] Dobrowsky P.H., Khan S., Khan W. (2017). Resistance of legionella and acanthamoeba mauritaniensis to heat treatment as determined by relative and quantitative polymerase chain reactions. Environ. Res..

[22] Alves Dde S., Moraes A.S., Alves L.M., Gurgel-Goncalves R., Lino Junior Rde S., Cuba-Cuba C.A., Vinaud M.C. (2016). Experimental infection of t4 acanthamoeba genotype determines the pathogenic potential. Parasitol. Res..

[23] Imayasu M., Tchedre K.T., Cavanagh H.D. (2013). Effects of multipurpose solutions on the viability and encystment of acanthamoeba determined by flow cytometry. Eye Contact Lens.

[24] Khunkitti W., Avery S.V., Lloyd D., Furr J.R., Russell A.D. (1997). Effects of biocides on acanthamoeba castellanii as measured by flow cytometry and plaque assay. J. Antimicrob. Chemother..

[25] Schober P., Boer C., Schwarte L.A. (2018). Correlation coefficients: Appropriate use and interpretation. Anesth. Analg..

[26] Xu M., Sivak J.G., McCanna D.J. (2013). Comparison of the effects of ophthalmic solutions on human corneal epithelial cells using fluorescent dyes. J. Ocul. Pharmacol. Ther..

[27] Lonnen J., Heaselgrave W., Nomachi M., Mori O., Santodomingo-Rubido J. (2010). Disinfection efficacy and encystment rate of soft contact lens multipurpose solutions against acanthamoeba. Eye Contact Lens.

[28] Padzik M., Chomicz L., Szaflik J.P., Chruscikowska A., Perkowski K., Szaflik J. (2014). In vitro effects of selected contact lens care solutions on acanthamoeba castellanii strains in poland. Exp. Parasitol..

[29] Martin-Navarro C.M., Lopez-Arencibia A., Sifaoui I., Reyes-Batlle M., Cabello-Vilchez A.M., Maciver S., Valladares B., Pinero J.E., Lorenzo-Morales J. (2014). Prestoblue(r) and alamarblue(r) are equally useful as agents to determine the viability of acanthamoeba trophozoites. Exp. Parasitol..

[30] Baig A.M., Iqbal J., Khan N.A. (2013). In vitro efficacies of clinically available drugs against growth and viability of an acanthamoeba castellanii keratitis isolate belonging to the t4 genotype. Antimicrob. Agents Chemother..

[31] Dive C., Watson J.V., Workman P. (1990). Multiparametric analysis of cell membrane permeability by two colour flow cytometry with complementary fluorescent probes. Cytometry.

[32] Hillmann F., Novohradská S., Mattern D.J., Forberger T., Heinekamp T., Westermann M., Winckler T., Brakhage A.A. (2015). Virulence determinants of the human pathogenic fungus aspergillus fumigatus protect against soil amoeba predation. Environ. Microbiol..

[33] Radosa S., Ferling I., Sprague J.L., Westermann M., Hillmann F. (2019). The different morphologies of yeast and filamentous fungi trigger distinct killing and feeding mechanisms in a fungivorous amoeba. Environ. Microbiol..

[34] Krämer C.E.M., Wiechert W., Kohlheyer D. (2016). Time-resolved, single-cell analysis of induced and programmed cell death via non-invasive propidium iodide and counterstain perfusion. Sci. Rep..

[35] Hellmold H., Teuteberg D., Tetens J., Blaschka C. (2020). 83 validation of propidium iodide dye for live-dead staining of bovine blastocysts: Preliminary results. Reprod. Fertil. Dev..

[36] Arnalich-Montiel F., Lumbreras-Fernández B., Martín-Navarro C.M., Valladares B., Lopez-Velez R., Morcillo-Laiz R., Lorenzo-Morales J. (2014). Influence of acanthamoeba genotype on clinical course and outcomes for patients with acanthamoeba keratitis in spain. J. Clin. Microbiol..

[37] Ledee D.R., Iovieno A., Miller D., Mandal N., Diaz M., Fell J., Fini M.E., Alfonso E.C. (2009). Molecular identification of t4 and t5 genotypes in isolates from acanthamoeba keratitis patients. J. Clin. Microbiol..

[38] Maghsood A.H., Sissons J., Rezaian M., Nolder D., Warhurst D., Khan N.A. (2005). Acanthamoeba genotype t4 from the uk and iran and isolation of the t2 genotype from clinical isolates. J. Med. Microbiol..

[39] Reed L.J., Muench H. (1938). A simple method of estimating fifty per cent endpoints. Am. J. Epidemiol..

[40] Vogt R.F., Marti G.E., Zenger V., Resch-Genger U. (2008). Quantitative fluorescence calibration: A tool for assessing the qualityof data obtained by fluorescence measurements. Standardization and Quality Assurance in Fluorescence Measurements Techniques.

